# Supplementary material for: Overlapping cell population expression profiling and regulatory inference in C. elegans
Source: BMC Genomics. 2016 Feb 29;17:159. doi: 10.1186/s12864-016-2482-z (PMC4772325; doi:10.1186/s12864-016-2482-z)
Supplement: Additional file 13: — Web supplement. (DOC 21 kb) [file 12864_2016_2482_MOESM13_ESM.zip › sortWeb/clusters/hier.300.clusters/286.html]

Cluster 286 

## Cluster 286

### Expression

| cnd-1 rep. 1 | cnd-1 rep. 2 | cnd-1 rep. 3 | pha-4 rep. 1 | pha-4 rep. 2 | pha-4 rep. 3 | ceh-27 | ceh-36 | ceh-6 | F21D5.9 | mir-57 | mls-2 | pal-1 | pros-1 | ttx-3 | unc-130 | hlh-16 | irx-1 | ceh-6 (+) hlh-16 (+) | ceh-6 (+) hlh-16 (-) | ceh-6 (-) hlh-16 (+) | cnd-1 singlets | pha-4 singlets | 0 | 60 | 120 | 150 | 180 | 240 | 330 | 390 | 420 | 480 | 540 | 570 | 600 | 630 | 660 | NAME | Functional description |
| --- | --- | --- | --- | --- | --- | --- | --- | --- | --- | --- | --- | --- | --- | --- | --- | --- | --- | --- | --- | --- | --- | --- | --- | --- | --- | --- | --- | --- | --- | --- | --- | --- | --- | --- | --- | --- | --- | --- | --- |
|  |  |  |  |  |  |  |  |  |  |  |  |  |  |  |  |  |  |  |  |  |  |  |  |  |  |  |  |  |  |  |  |  |  |  |  |  |  | C43E11.5 |  |
|  |  |  |  |  |  |  |  |  |  |  |  |  |  |  |  |  |  |  |  |  |  |  |  |  |  |  |  |  |  |  |  |  |  |  |  |  |  | *srh-82* | Serpentine Receptor, class H |
|  |  |  |  |  |  |  |  |  |  |  |  |  |  |  |  |  |  |  |  |  |  |  |  |  |  |  |  |  |  |  |  |  |  |  |  |  |  | *fbxb-69* | F-box B protein |
|  |  |  |  |  |  |  |  |  |  |  |  |  |  |  |  |  |  |  |  |  |  |  |  |  |  |  |  |  |  |  |  |  |  |  |  |  |  | *jbts-14* | JouBerT Syndrome homolog |
|  |  |  |  |  |  |  |  |  |  |  |  |  |  |  |  |  |  |  |  |  |  |  |  |  |  |  |  |  |  |  |  |  |  |  |  |  |  | C03C10.9 |  |
|  |  |  |  |  |  |  |  |  |  |  |  |  |  |  |  |  |  |  |  |  |  |  |  |  |  |  |  |  |  |  |  |  |  |  |  |  |  | C03C10.5 |  |
|  |  |  |  |  |  |  |  |  |  |  |  |  |  |  |  |  |  |  |  |  |  |  |  |  |  |  |  |  |  |  |  |  |  |  |  |  |  | *arl-6* | ARF-Like |
|  |  |  |  |  |  |  |  |  |  |  |  |  |  |  |  |  |  |  |  |  |  |  |  |  |  |  |  |  |  |  |  |  |  |  |  |  |  | K07C11.10 |  |
|  |  |  |  |  |  |  |  |  |  |  |  |  |  |  |  |  |  |  |  |  |  |  |  |  |  |  |  |  |  |  |  |  |  |  |  |  |  | T26A8.3 |  |
|  |  |  |  |  |  |  |  |  |  |  |  |  |  |  |  |  |  |  |  |  |  |  |  |  |  |  |  |  |  |  |  |  |  |  |  |  |  | ZK863.1 |  |
|  |  |  |  |  |  |  |  |  |  |  |  |  |  |  |  |  |  |  |  |  |  |  |  |  |  |  |  |  |  |  |  |  |  |  |  |  |  | Y23H5B.1 |  |
|  |  |  |  |  |  |  |  |  |  |  |  |  |  |  |  |  |  |  |  |  |  |  |  |  |  |  |  |  |  |  |  |  |  |  |  |  |  | *vab-3* | Variable ABnormal morphology |
|  |  |  |  |  |  |  |  |  |  |  |  |  |  |  |  |  |  |  |  |  |  |  |  |  |  |  |  |  |  |  |  |  |  |  |  |  |  | ZK973.9 |  |
|  |  |  |  |  |  |  |  |  |  |  |  |  |  |  |  |  |  |  |  |  |  |  |  |  |  |  |  |  |  |  |  |  |  |  |  |  |  | K02B12.2 |  |
|  |  |  |  |  |  |  |  |  |  |  |  |  |  |  |  |  |  |  |  |  |  |  |  |  |  |  |  |  |  |  |  |  |  |  |  |  |  | *bra-1* | BMP Receptor Associated protein family |
|  |  |  |  |  |  |  |  |  |  |  |  |  |  |  |  |  |  |  |  |  |  |  |  |  |  |  |  |  |  |  |  |  |  |  |  |  |  | *fbxb-70* | F-box B protein |
|  |  |  |  |  |  |  |  |  |  |  |  |  |  |  |  |  |  |  |  |  |  |  |  |  |  |  |  |  |  |  |  |  |  |  |  |  |  | *math-34* | MATH (meprin-associated Traf homology) domain containing |
|  |  |  |  |  |  |  |  |  |  |  |  |  |  |  |  |  |  |  |  |  |  |  |  |  |  |  |  |  |  |  |  |  |  |  |  |  |  | K02E7.5 |  |
|  |  |  |  |  |  |  |  |  |  |  |  |  |  |  |  |  |  |  |  |  |  |  |  |  |  |  |  |  |  |  |  |  |  |  |  |  |  | *dro-1* | DR1 (One) transcription factor family |
|  |  |  |  |  |  |  |  |  |  |  |  |  |  |  |  |  |  |  |  |  |  |  |  |  |  |  |  |  |  |  |  |  |  |  |  |  |  | *nud-1* | Aspergillus NUclear Division related |
|  |  |  |  |  |  |  |  |  |  |  |  |  |  |  |  |  |  |  |  |  |  |  |  |  |  |  |  |  |  |  |  |  |  |  |  |  |  | C04A11.2 |  |
|  |  |  |  |  |  |  |  |  |  |  |  |  |  |  |  |  |  |  |  |  |  |  |  |  |  |  |  |  |  |  |  |  |  |  |  |  |  | *pho-5* | intestinal acid PHOsphatase |
|  |  |  |  |  |  |  |  |  |  |  |  |  |  |  |  |  |  |  |  |  |  |  |  |  |  |  |  |  |  |  |  |  |  |  |  |  |  | *unc-129* | UNCoordinated |
|  |  |  |  |  |  |  |  |  |  |  |  |  |  |  |  |  |  |  |  |  |  |  |  |  |  |  |  |  |  |  |  |  |  |  |  |  |  | F41G4.7 |  |
|  |  |  |  |  |  |  |  |  |  |  |  |  |  |  |  |  |  |  |  |  |  |  |  |  |  |  |  |  |  |  |  |  |  |  |  |  |  | *tbb-4* | TuBulin, Beta |
|  |  |  |  |  |  |  |  |  |  |  |  |  |  |  |  |  |  |  |  |  |  |  |  |  |  |  |  |  |  |  |  |  |  |  |  |  |  | *cdh-12* | CaDHerin family |
|  |  |  |  |  |  |  |  |  |  |  |  |  |  |  |  |  |  |  |  |  |  |  |  |  |  |  |  |  |  |  |  |  |  |  |  |  |  | Y38H6C.14 |  |
|  |  |  |  |  |  |  |  |  |  |  |  |  |  |  |  |  |  |  |  |  |  |  |  |  |  |  |  |  |  |  |  |  |  |  |  |  |  | W07G1.1 |  |
|  |  |  |  |  |  |  |  |  |  |  |  |  |  |  |  |  |  |  |  |  |  |  |  |  |  |  |  |  |  |  |  |  |  |  |  |  |  | *sri-48* | Serpentine Receptor, class I |
|  |  |  |  |  |  |  |  |  |  |  |  |  |  |  |  |  |  |  |  |  |  |  |  |  |  |  |  |  |  |  |  |  |  |  |  |  |  | C16C10.9 |  |
|  |  |  |  |  |  |  |  |  |  |  |  |  |  |  |  |  |  |  |  |  |  |  |  |  |  |  |  |  |  |  |  |  |  |  |  |  |  | *col-36* | COLlagen |
|  |  |  |  |  |  |  |  |  |  |  |  |  |  |  |  |  |  |  |  |  |  |  |  |  |  |  |  |  |  |  |  |  |  |  |  |  |  | Y22D7AL.4 |  |
|  |  |  |  |  |  |  |  |  |  |  |  |  |  |  |  |  |  |  |  |  |  |  |  |  |  |  |  |  |  |  |  |  |  |  |  |  |  | T03G11.3 |  |
|  |  |  |  |  |  |  |  |  |  |  |  |  |  |  |  |  |  |  |  |  |  |  |  |  |  |  |  |  |  |  |  |  |  |  |  |  |  | F27C1.13 |  |
|  |  |  |  |  |  |  |  |  |  |  |  |  |  |  |  |  |  |  |  |  |  |  |  |  |  |  |  |  |  |  |  |  |  |  |  |  |  | *irld-2* | Insulin/EGF-Receptor L Domain protein |
|  |  |  |  |  |  |  |  |  |  |  |  |  |  |  |  |  |  |  |  |  |  |  |  |  |  |  |  |  |  |  |  |  |  |  |  |  |  | *fbxa-170* | F-box A protein |
|  |  |  |  |  |  |  |  |  |  |  |  |  |  |  |  |  |  |  |  |  |  |  |  |  |  |  |  |  |  |  |  |  |  |  |  |  |  | *fozi-1* | FOrmin-homology and ZInc finger domains |
|  |  |  |  |  |  |  |  |  |  |  |  |  |  |  |  |  |  |  |  |  |  |  |  |  |  |  |  |  |  |  |  |  |  |  |  |  |  | *gpa-10* | G Protein, Alpha subunit |
|  |  |  |  |  |  |  |  |  |  |  |  |  |  |  |  |  |  |  |  |  |  |  |  |  |  |  |  |  |  |  |  |  |  |  |  |  |  | F49D11.2 |  |
|  |  |  |  |  |  |  |  |  |  |  |  |  |  |  |  |  |  |  |  |  |  |  |  |  |  |  |  |  |  |  |  |  |  |  |  |  |  | K08C7.4 |  |
|  |  |  |  |  |  |  |  |  |  |  |  |  |  |  |  |  |  |  |  |  |  |  |  |  |  |  |  |  |  |  |  |  |  |  |  |  |  | Y113G7A.14 |  |
|  |  |  |  |  |  |  |  |  |  |  |  |  |  |  |  |  |  |  |  |  |  |  |  |  |  |  |  |  |  |  |  |  |  |  |  |  |  | C44B11.1 |  |
|  |  |  |  |  |  |  |  |  |  |  |  |  |  |  |  |  |  |  |  |  |  |  |  |  |  |  |  |  |  |  |  |  |  |  |  |  |  | R01H10.5 |  |
|  |  |  |  |  |  |  |  |  |  |  |  |  |  |  |  |  |  |  |  |  |  |  |  |  |  |  |  |  |  |  |  |  |  |  |  |  |  | C27A2.8 |  |
|  |  |  |  |  |  |  |  |  |  |  |  |  |  |  |  |  |  |  |  |  |  |  |  |  |  |  |  |  |  |  |  |  |  |  |  |  |  | C33A12.4 |  |
|  |  |  |  |  |  |  |  |  |  |  |  |  |  |  |  |  |  |  |  |  |  |  |  |  |  |  |  |  |  |  |  |  |  |  |  |  |  | Y53C10A.5 |  |
|  |  |  |  |  |  |  |  |  |  |  |  |  |  |  |  |  |  |  |  |  |  |  |  |  |  |  |  |  |  |  |  |  |  |  |  |  |  | *fkh-8* | ForKHead transcription factor family |
|  |  |  |  |  |  |  |  |  |  |  |  |  |  |  |  |  |  |  |  |  |  |  |  |  |  |  |  |  |  |  |  |  |  |  |  |  |  | *del-2* | DEgenerin Like |
|  |  |  |  |  |  |  |  |  |  |  |  |  |  |  |  |  |  |  |  |  |  |  |  |  |  |  |  |  |  |  |  |  |  |  |  |  |  | *gasr-8* |  |
|  |  |  |  |  |  |  |  |  |  |  |  |  |  |  |  |  |  |  |  |  |  |  |  |  |  |  |  |  |  |  |  |  |  |  |  |  |  | *che-2* | abnormal CHEmotaxis |
|  |  |  |  |  |  |  |  |  |  |  |  |  |  |  |  |  |  |  |  |  |  |  |  |  |  |  |  |  |  |  |  |  |  |  |  |  |  | F48E3.9 |  |
|  |  |  |  |  |  |  |  |  |  |  |  |  |  |  |  |  |  |  |  |  |  |  |  |  |  |  |  |  |  |  |  |  |  |  |  |  |  | *daf-10* | abnormal DAuer Formation |
|  |  |  |  |  |  |  |  |  |  |  |  |  |  |  |  |  |  |  |  |  |  |  |  |  |  |  |  |  |  |  |  |  |  |  |  |  |  | *nphp-1* | NePHronoPhthisis (human kidney disease) homolog |
|  |  |  |  |  |  |  |  |  |  |  |  |  |  |  |  |  |  |  |  |  |  |  |  |  |  |  |  |  |  |  |  |  |  |  |  |  |  | *dyf-2* | abnormal DYe Filling |
|  |  |  |  |  |  |  |  |  |  |  |  |  |  |  |  |  |  |  |  |  |  |  |  |  |  |  |  |  |  |  |  |  |  |  |  |  |  | *ift-81* | IFT (Chlamydomonas IntraFlagellar Transport) homolog |
|  |  |  |  |  |  |  |  |  |  |  |  |  |  |  |  |  |  |  |  |  |  |  |  |  |  |  |  |  |  |  |  |  |  |  |  |  |  | *osm-5* | OSMotic avoidance abnormal |
|  |  |  |  |  |  |  |  |  |  |  |  |  |  |  |  |  |  |  |  |  |  |  |  |  |  |  |  |  |  |  |  |  |  |  |  |  |  | *mks-3* | MecKel-Gruber Syndrome (MKS) homolog |
|  |  |  |  |  |  |  |  |  |  |  |  |  |  |  |  |  |  |  |  |  |  |  |  |  |  |  |  |  |  |  |  |  |  |  |  |  |  | *nphp-4* | NePHronoPhthisis (human kidney disease) homolog |
|  |  |  |  |  |  |  |  |  |  |  |  |  |  |  |  |  |  |  |  |  |  |  |  |  |  |  |  |  |  |  |  |  |  |  |  |  |  | *mks-5* | MecKel-Gruber Syndrome (MKS) homolog |
|  |  |  |  |  |  |  |  |  |  |  |  |  |  |  |  |  |  |  |  |  |  |  |  |  |  |  |  |  |  |  |  |  |  |  |  |  |  | Y47G6A.17 |  |
|  |  |  |  |  |  |  |  |  |  |  |  |  |  |  |  |  |  |  |  |  |  |  |  |  |  |  |  |  |  |  |  |  |  |  |  |  |  | *ifta-1* | IntraFlagellar Transport Associated |
|  |  |  |  |  |  |  |  |  |  |  |  |  |  |  |  |  |  |  |  |  |  |  |  |  |  |  |  |  |  |  |  |  |  |  |  |  |  | *dyf-13* | abnormal DYe Filling |
|  |  |  |  |  |  |  |  |  |  |  |  |  |  |  |  |  |  |  |  |  |  |  |  |  |  |  |  |  |  |  |  |  |  |  |  |  |  | Y57G11C.32 |  |
|  |  |  |  |  |  |  |  |  |  |  |  |  |  |  |  |  |  |  |  |  |  |  |  |  |  |  |  |  |  |  |  |  |  |  |  |  |  | *tctn-1* | TeCToNic (vertebrate ciliogenesis factor) homolog |
|  |  |  |  |  |  |  |  |  |  |  |  |  |  |  |  |  |  |  |  |  |  |  |  |  |  |  |  |  |  |  |  |  |  |  |  |  |  | C43H6.6 |  |
|  |  |  |  |  |  |  |  |  |  |  |  |  |  |  |  |  |  |  |  |  |  |  |  |  |  |  |  |  |  |  |  |  |  |  |  |  |  | C34D4.1 |  |
|  |  |  |  |  |  |  |  |  |  |  |  |  |  |  |  |  |  |  |  |  |  |  |  |  |  |  |  |  |  |  |  |  |  |  |  |  |  | *mks-6* | MecKel-Gruber Syndrome (MKS) homolog |
|  |  |  |  |  |  |  |  |  |  |  |  |  |  |  |  |  |  |  |  |  |  |  |  |  |  |  |  |  |  |  |  |  |  |  |  |  |  | *dyf-5* | abnormal DYe Filling |
|  |  |  |  |  |  |  |  |  |  |  |  |  |  |  |  |  |  |  |  |  |  |  |  |  |  |  |  |  |  |  |  |  |  |  |  |  |  | *dyf-11* | abnormal DYe Filling |
|  |  |  |  |  |  |  |  |  |  |  |  |  |  |  |  |  |  |  |  |  |  |  |  |  |  |  |  |  |  |  |  |  |  |  |  |  |  | *bbs-2* | BBS (Bardet-Biedl Syndrome) protein |
|  |  |  |  |  |  |  |  |  |  |  |  |  |  |  |  |  |  |  |  |  |  |  |  |  |  |  |  |  |  |  |  |  |  |  |  |  |  | K04F10.2 |  |
|  |  |  |  |  |  |  |  |  |  |  |  |  |  |  |  |  |  |  |  |  |  |  |  |  |  |  |  |  |  |  |  |  |  |  |  |  |  | *bbs-9* | BBS (Bardet-Biedl Syndrome) protein |
|  |  |  |  |  |  |  |  |  |  |  |  |  |  |  |  |  |  |  |  |  |  |  |  |  |  |  |  |  |  |  |  |  |  |  |  |  |  | T02G5.3 |  |
|  |  |  |  |  |  |  |  |  |  |  |  |  |  |  |  |  |  |  |  |  |  |  |  |  |  |  |  |  |  |  |  |  |  |  |  |  |  | *ceh-57* | C. Elegans Homeobox |
|  |  |  |  |  |  |  |  |  |  |  |  |  |  |  |  |  |  |  |  |  |  |  |  |  |  |  |  |  |  |  |  |  |  |  |  |  |  | T02G5.2 |  |
|  |  |  |  |  |  |  |  |  |  |  |  |  |  |  |  |  |  |  |  |  |  |  |  |  |  |  |  |  |  |  |  |  |  |  |  |  |  | T02G5.1 |  |
|  |  |  |  |  |  |  |  |  |  |  |  |  |  |  |  |  |  |  |  |  |  |  |  |  |  |  |  |  |  |  |  |  |  |  |  |  |  | ZK682.7 |  |
|  |  |  |  |  |  |  |  |  |  |  |  |  |  |  |  |  |  |  |  |  |  |  |  |  |  |  |  |  |  |  |  |  |  |  |  |  |  | ZC13.2 |  |
|  |  |  |  |  |  |  |  |  |  |  |  |  |  |  |  |  |  |  |  |  |  |  |  |  |  |  |  |  |  |  |  |  |  |  |  |  |  | ZK813.5 |  |
|  |  |  |  |  |  |  |  |  |  |  |  |  |  |  |  |  |  |  |  |  |  |  |  |  |  |  |  |  |  |  |  |  |  |  |  |  |  | F59G1.4 |  |
|  |  |  |  |  |  |  |  |  |  |  |  |  |  |  |  |  |  |  |  |  |  |  |  |  |  |  |  |  |  |  |  |  |  |  |  |  |  | *dyf-18* | abnormal DYe Filling |
|  |  |  |  |  |  |  |  |  |  |  |  |  |  |  |  |  |  |  |  |  |  |  |  |  |  |  |  |  |  |  |  |  |  |  |  |  |  | C05D10.2 |  |
|  |  |  |  |  |  |  |  |  |  |  |  |  |  |  |  |  |  |  |  |  |  |  |  |  |  |  |  |  |  |  |  |  |  |  |  |  |  | *dyf-1* | abnormal DYe Filling |
|  |  |  |  |  |  |  |  |  |  |  |  |  |  |  |  |  |  |  |  |  |  |  |  |  |  |  |  |  |  |  |  |  |  |  |  |  |  | F59B10.2 |  |
|  |  |  |  |  |  |  |  |  |  |  |  |  |  |  |  |  |  |  |  |  |  |  |  |  |  |  |  |  |  |  |  |  |  |  |  |  |  | *bbs-4* | BBS (Bardet-Biedl Syndrome) protein |
|  |  |  |  |  |  |  |  |  |  |  |  |  |  |  |  |  |  |  |  |  |  |  |  |  |  |  |  |  |  |  |  |  |  |  |  |  |  | *che-13* | abnormal CHEmotaxis |
|  |  |  |  |  |  |  |  |  |  |  |  |  |  |  |  |  |  |  |  |  |  |  |  |  |  |  |  |  |  |  |  |  |  |  |  |  |  | *osm-6* | OSMotic avoidance abnormal |
|  |  |  |  |  |  |  |  |  |  |  |  |  |  |  |  |  |  |  |  |  |  |  |  |  |  |  |  |  |  |  |  |  |  |  |  |  |  | R07E5.17 |  |
|  |  |  |  |  |  |  |  |  |  |  |  |  |  |  |  |  |  |  |  |  |  |  |  |  |  |  |  |  |  |  |  |  |  |  |  |  |  | T26A8.2 |  |
|  |  |  |  |  |  |  |  |  |  |  |  |  |  |  |  |  |  |  |  |  |  |  |  |  |  |  |  |  |  |  |  |  |  |  |  |  |  | *rab-28* | RAB family |
|  |  |  |  |  |  |  |  |  |  |  |  |  |  |  |  |  |  |  |  |  |  |  |  |  |  |  |  |  |  |  |  |  |  |  |  |  |  | *arl-13* | ARF-Like |
|  |  |  |  |  |  |  |  |  |  |  |  |  |  |  |  |  |  |  |  |  |  |  |  |  |  |  |  |  |  |  |  |  |  |  |  |  |  | *bbs-5* | BBS (Bardet-Biedl Syndrome) protein |
|  |  |  |  |  |  |  |  |  |  |  |  |  |  |  |  |  |  |  |  |  |  |  |  |  |  |  |  |  |  |  |  |  |  |  |  |  |  | *mksr-2* | MecKel Syndrome 1 (MKS1)-Related |
|  |  |  |  |  |  |  |  |  |  |  |  |  |  |  |  |  |  |  |  |  |  |  |  |  |  |  |  |  |  |  |  |  |  |  |  |  |  | *ift-20* | IFT (Chlamydomonas IntraFlagellar Transport) homolog |
|  |  |  |  |  |  |  |  |  |  |  |  |  |  |  |  |  |  |  |  |  |  |  |  |  |  |  |  |  |  |  |  |  |  |  |  |  |  | *ifta-2* | IntraFlagellar Transport Associated |
|  |  |  |  |  |  |  |  |  |  |  |  |  |  |  |  |  |  |  |  |  |  |  |  |  |  |  |  |  |  |  |  |  |  |  |  |  |  | Y50C1A.2 |  |
|  |  |  |  |  |  |  |  |  |  |  |  |  |  |  |  |  |  |  |  |  |  |  |  |  |  |  |  |  |  |  |  |  |  |  |  |  |  | *xbx-1* | X-BoX promoter element regulated |
|  |  |  |  |  |  |  |  |  |  |  |  |  |  |  |  |  |  |  |  |  |  |  |  |  |  |  |  |  |  |  |  |  |  |  |  |  |  | T05A8.5 |  |
|  |  |  |  |  |  |  |  |  |  |  |  |  |  |  |  |  |  |  |  |  |  |  |  |  |  |  |  |  |  |  |  |  |  |  |  |  |  | *dyf-17* | abnormal DYe Filling |
|  |  |  |  |  |  |  |  |  |  |  |  |  |  |  |  |  |  |  |  |  |  |  |  |  |  |  |  |  |  |  |  |  |  |  |  |  |  | *bbs-1* | BBS (Bardet-Biedl Syndrome) protein |
|  |  |  |  |  |  |  |  |  |  |  |  |  |  |  |  |  |  |  |  |  |  |  |  |  |  |  |  |  |  |  |  |  |  |  |  |  |  | *dyf-3* | abnormal DYe Filling |
|  |  |  |  |  |  |  |  |  |  |  |  |  |  |  |  |  |  |  |  |  |  |  |  |  |  |  |  |  |  |  |  |  |  |  |  |  |  | *che-3* | abnormal CHEmotaxis |
|  |  |  |  |  |  |  |  |  |  |  |  |  |  |  |  |  |  |  |  |  |  |  |  |  |  |  |  |  |  |  |  |  |  |  |  |  |  | *che-11* | abnormal CHEmotaxis |
|  |  |  |  |  |  |  |  |  |  |  |  |  |  |  |  |  |  |  |  |  |  |  |  |  |  |  |  |  |  |  |  |  |  |  |  |  |  | C27F2.1 |  |
|  |  |  |  |  |  |  |  |  |  |  |  |  |  |  |  |  |  |  |  |  |  |  |  |  |  |  |  |  |  |  |  |  |  |  |  |  |  | ZK328.7 |  |
|  |  |  |  |  |  |  |  |  |  |  |  |  |  |  |  |  |  |  |  |  |  |  |  |  |  |  |  |  |  |  |  |  |  |  |  |  |  | *osm-12* | OSMotic avoidance abnormal |
|  |  |  |  |  |  |  |  |  |  |  |  |  |  |  |  |  |  |  |  |  |  |  |  |  |  |  |  |  |  |  |  |  |  |  |  |  |  | *osm-1* | OSMotic avoidance abnormal |
|  |  |  |  |  |  |  |  |  |  |  |  |  |  |  |  |  |  |  |  |  |  |  |  |  |  |  |  |  |  |  |  |  |  |  |  |  |  | F01E11.3 |  |
|  |  |  |  |  |  |  |  |  |  |  |  |  |  |  |  |  |  |  |  |  |  |  |  |  |  |  |  |  |  |  |  |  |  |  |  |  |  | *mks-1* | MecKel-Gruber Syndrome (MKS) homolog |
|  |  |  |  |  |  |  |  |  |  |  |  |  |  |  |  |  |  |  |  |  |  |  |  |  |  |  |  |  |  |  |  |  |  |  |  |  |  | *lgc-20* | Ligand-Gated ion Channel |
|  |  |  |  |  |  |  |  |  |  |  |  |  |  |  |  |  |  |  |  |  |  |  |  |  |  |  |  |  |  |  |  |  |  |  |  |  |  | *bbs-8* | BBS (Bardet-Biedl Syndrome) protein |
|  |  |  |  |  |  |  |  |  |  |  |  |  |  |  |  |  |  |  |  |  |  |  |  |  |  |  |  |  |  |  |  |  |  |  |  |  |  | Y43F8C.4 |  |
|  |  |  |  |  |  |  |  |  |  |  |  |  |  |  |  |  |  |  |  |  |  |  |  |  |  |  |  |  |  |  |  |  |  |  |  |  |  | *ift-74* | IFT (Chlamydomonas IntraFlagellar Transport) homolog |
|  |  |  |  |  |  |  |  |  |  |  |  |  |  |  |  |  |  |  |  |  |  |  |  |  |  |  |  |  |  |  |  |  |  |  |  |  |  | *tub-1* | TUBby-related |
|  |  |  |  |  |  |  |  |  |  |  |  |  |  |  |  |  |  |  |  |  |  |  |  |  |  |  |  |  |  |  |  |  |  |  |  |  |  | *mksr-1* | MecKel Syndrome 1 (MKS1)-Related |
|  |  |  |  |  |  |  |  |  |  |  |  |  |  |  |  |  |  |  |  |  |  |  |  |  |  |  |  |  |  |  |  |  |  |  |  |  |  | F32D8.1 |  |
|  |  |  |  |  |  |  |  |  |  |  |  |  |  |  |  |  |  |  |  |  |  |  |  |  |  |  |  |  |  |  |  |  |  |  |  |  |  | Y42A5A.4 |  |
|  |  |  |  |  |  |  |  |  |  |  |  |  |  |  |  |  |  |  |  |  |  |  |  |  |  |  |  |  |  |  |  |  |  |  |  |  |  | F32B4.1 |  |
|  |  |  |  |  |  |  |  |  |  |  |  |  |  |  |  |  |  |  |  |  |  |  |  |  |  |  |  |  |  |  |  |  |  |  |  |  |  | F02D8.4 |  |
|  |  |  |  |  |  |  |  |  |  |  |  |  |  |  |  |  |  |  |  |  |  |  |  |  |  |  |  |  |  |  |  |  |  |  |  |  |  | *gpa-14* | G Protein, Alpha subunit |
|  |  |  |  |  |  |  |  |  |  |  |  |  |  |  |  |  |  |  |  |  |  |  |  |  |  |  |  |  |  |  |  |  |  |  |  |  |  | F14D7.8 |  |
|  |  |  |  |  |  |  |  |  |  |  |  |  |  |  |  |  |  |  |  |  |  |  |  |  |  |  |  |  |  |  |  |  |  |  |  |  |  | C36C9.5 |  |
|  |  |  |  |  |  |  |  |  |  |  |  |  |  |  |  |  |  |  |  |  |  |  |  |  |  |  |  |  |  |  |  |  |  |  |  |  |  | R10D12.10 |  |
|  |  |  |  |  |  |  |  |  |  |  |  |  |  |  |  |  |  |  |  |  |  |  |  |  |  |  |  |  |  |  |  |  |  |  |  |  |  | *osm-3* | OSMotic avoidance abnormal |
|  |  |  |  |  |  |  |  |  |  |  |  |  |  |  |  |  |  |  |  |  |  |  |  |  |  |  |  |  |  |  |  |  |  |  |  |  |  | *nekl-1* | NEK (NEver in mitosis Kinase) Like |
|  |  |  |  |  |  |  |  |  |  |  |  |  |  |  |  |  |  |  |  |  |  |  |  |  |  |  |  |  |  |  |  |  |  |  |  |  |  | *cng-2* | Cyclic Nucleotide Gated channel |
|  |  |  |  |  |  |  |  |  |  |  |  |  |  |  |  |  |  |  |  |  |  |  |  |  |  |  |  |  |  |  |  |  |  |  |  |  |  | C56E6.4 |  |
|  |  |  |  |  |  |  |  |  |  |  |  |  |  |  |  |  |  |  |  |  |  |  |  |  |  |  |  |  |  |  |  |  |  |  |  |  |  | *pnc-1* | Pyrazinamidase and NiCotinamidase |
|  |  |  |  |  |  |  |  |  |  |  |  |  |  |  |  |  |  |  |  |  |  |  |  |  |  |  |  |  |  |  |  |  |  |  |  |  |  | *nhr-277* | Nuclear Hormone Receptor family |
|  |  |  |  |  |  |  |  |  |  |  |  |  |  |  |  |  |  |  |  |  |  |  |  |  |  |  |  |  |  |  |  |  |  |  |  |  |  | *tba-5* | TuBulin, Alpha |
|  |  |  |  |  |  |  |  |  |  |  |  |  |  |  |  |  |  |  |  |  |  |  |  |  |  |  |  |  |  |  |  |  |  |  |  |  |  | *gpa-3* | G Protein, Alpha subunit |
|  |  |  |  |  |  |  |  |  |  |  |  |  |  |  |  |  |  |  |  |  |  |  |  |  |  |  |  |  |  |  |  |  |  |  |  |  |  | *klp-13* | Kinesin-Like Protein |
|  |  |  |  |  |  |  |  |  |  |  |  |  |  |  |  |  |  |  |  |  |  |  |  |  |  |  |  |  |  |  |  |  |  |  |  |  |  | Y38H6C.24 |  |
|  |  |  |  |  |  |  |  |  |  |  |  |  |  |  |  |  |  |  |  |  |  |  |  |  |  |  |  |  |  |  |  |  |  |  |  |  |  | F53G12.4 |  |
|  |  |  |  |  |  |  |  |  |  |  |  |  |  |  |  |  |  |  |  |  |  |  |  |  |  |  |  |  |  |  |  |  |  |  |  |  |  | *sri-47* | Serpentine Receptor, class I |
|  |  |  |  |  |  |  |  |  |  |  |  |  |  |  |  |  |  |  |  |  |  |  |  |  |  |  |  |  |  |  |  |  |  |  |  |  |  | Y53G8AL.1 |  |
|  |  |  |  |  |  |  |  |  |  |  |  |  |  |  |  |  |  |  |  |  |  |  |  |  |  |  |  |  |  |  |  |  |  |  |  |  |  | *anoh-1* | ANOctamin (calcium-activated chloride channel) Homolog |
|  |  |  |  |  |  |  |  |  |  |  |  |  |  |  |  |  |  |  |  |  |  |  |  |  |  |  |  |  |  |  |  |  |  |  |  |  |  | H41C03.3 |  |
|  |  |  |  |  |  |  |  |  |  |  |  |  |  |  |  |  |  |  |  |  |  |  |  |  |  |  |  |  |  |  |  |  |  |  |  |  |  | T05C12.8 |  |
|  |  |  |  |  |  |  |  |  |  |  |  |  |  |  |  |  |  |  |  |  |  |  |  |  |  |  |  |  |  |  |  |  |  |  |  |  |  | F09E5.12 |  |
|  |  |  |  |  |  |  |  |  |  |  |  |  |  |  |  |  |  |  |  |  |  |  |  |  |  |  |  |  |  |  |  |  |  |  |  |  |  | F44G4.7 |  |
|  |  |  |  |  |  |  |  |  |  |  |  |  |  |  |  |  |  |  |  |  |  |  |  |  |  |  |  |  |  |  |  |  |  |  |  |  |  | D1007.3 |  |
|  |  |  |  |  |  |  |  |  |  |  |  |  |  |  |  |  |  |  |  |  |  |  |  |  |  |  |  |  |  |  |  |  |  |  |  |  |  | F38B7.10 |  |
|  |  |  |  |  |  |  |  |  |  |  |  |  |  |  |  |  |  |  |  |  |  |  |  |  |  |  |  |  |  |  |  |  |  |  |  |  |  | *nhr-258* | Nuclear Hormone Receptor family |
|  |  |  |  |  |  |  |  |  |  |  |  |  |  |  |  |  |  |  |  |  |  |  |  |  |  |  |  |  |  |  |  |  |  |  |  |  |  | *gpa-18* | G Protein, Alpha subunit |
|  |  |  |  |  |  |  |  |  |  |  |  |  |  |  |  |  |  |  |  |  |  |  |  |  |  |  |  |  |  |  |  |  |  |  |  |  |  | *nhr-51* | Nuclear Hormone Receptor family |
|  |  |  |  |  |  |  |  |  |  |  |  |  |  |  |  |  |  |  |  |  |  |  |  |  |  |  |  |  |  |  |  |  |  |  |  |  |  | *nhr-52* | Nuclear Hormone Receptor family |
|  |  |  |  |  |  |  |  |  |  |  |  |  |  |  |  |  |  |  |  |  |  |  |  |  |  |  |  |  |  |  |  |  |  |  |  |  |  | *dhhc-11* | DHHC-types zinc finger protein |
|  |  |  |  |  |  |  |  |  |  |  |  |  |  |  |  |  |  |  |  |  |  |  |  |  |  |  |  |  |  |  |  |  |  |  |  |  |  | *fbxa-81* | F-box A protein |
|  |  |  |  |  |  |  |  |  |  |  |  |  |  |  |  |  |  |  |  |  |  |  |  |  |  |  |  |  |  |  |  |  |  |  |  |  |  | *rmd-3* | Regulator of Microtubule Dynamics |
|  |  |  |  |  |  |  |  |  |  |  |  |  |  |  |  |  |  |  |  |  |  |  |  |  |  |  |  |  |  |  |  |  |  |  |  |  |  | F38B7.11 |  |
|  |  |  |  |  |  |  |  |  |  |  |  |  |  |  |  |  |  |  |  |  |  |  |  |  |  |  |  |  |  |  |  |  |  |  |  |  |  | M04C9.4 |  |
|  |  |  |  |  |  |  |  |  |  |  |  |  |  |  |  |  |  |  |  |  |  |  |  |  |  |  |  |  |  |  |  |  |  |  |  |  |  | *osta-1* | Organic Solute Transporter Alpha-like protein |
|  |  |  |  |  |  |  |  |  |  |  |  |  |  |  |  |  |  |  |  |  |  |  |  |  |  |  |  |  |  |  |  |  |  |  |  |  |  | Y102A11A.9 |  |
|  |  |  |  |  |  |  |  |  |  |  |  |  |  |  |  |  |  |  |  |  |  |  |  |  |  |  |  |  |  |  |  |  |  |  |  |  |  | *srd-74* | Serpentine Receptor, class D (delta) |
|  |  |  |  |  |  |  |  |  |  |  |  |  |  |  |  |  |  |  |  |  |  |  |  |  |  |  |  |  |  |  |  |  |  |  |  |  |  | T03G11.20 |  |
|  |  |  |  |  |  |  |  |  |  |  |  |  |  |  |  |  |  |  |  |  |  |  |  |  |  |  |  |  |  |  |  |  |  |  |  |  |  | Y105E8A.47 |  |
|  |  |  |  |  |  |  |  |  |  |  |  |  |  |  |  |  |  |  |  |  |  |  |  |  |  |  |  |  |  |  |  |  |  |  |  |  |  | *kin-6* | protein KINase |
|  |  |  |  |  |  |  |  |  |  |  |  |  |  |  |  |  |  |  |  |  |  |  |  |  |  |  |  |  |  |  |  |  |  |  |  |  |  | *sqt-1* | SQuaT |
|  |  |  |  |  |  |  |  |  |  |  |  |  |  |  |  |  |  |  |  |  |  |  |  |  |  |  |  |  |  |  |  |  |  |  |  |  |  | F52D10.12 |  |
|  |  |  |  |  |  |  |  |  |  |  |  |  |  |  |  |  |  |  |  |  |  |  |  |  |  |  |  |  |  |  |  |  |  |  |  |  |  | *srh-221* | Serpentine Receptor, class H |
|  |  |  |  |  |  |  |  |  |  |  |  |  |  |  |  |  |  |  |  |  |  |  |  |  |  |  |  |  |  |  |  |  |  |  |  |  |  | Y80D3A.14 |  |
|  |  |  |  |  |  |  |  |  |  |  |  |  |  |  |  |  |  |  |  |  |  |  |  |  |  |  |  |  |  |  |  |  |  |  |  |  |  | C04D8.4 |  |
|  |  |  |  |  |  |  |  |  |  |  |  |  |  |  |  |  |  |  |  |  |  |  |  |  |  |  |  |  |  |  |  |  |  |  |  |  |  | C01G12.9 |  |
|  |  |  |  |  |  |  |  |  |  |  |  |  |  |  |  |  |  |  |  |  |  |  |  |  |  |  |  |  |  |  |  |  |  |  |  |  |  | *duox-2* | DUal OXidase |
|  |  |  |  |  |  |  |  |  |  |  |  |  |  |  |  |  |  |  |  |  |  |  |  |  |  |  |  |  |  |  |  |  |  |  |  |  |  | R06B10.2 |  |
|  |  |  |  |  |  |  |  |  |  |  |  |  |  |  |  |  |  |  |  |  |  |  |  |  |  |  |  |  |  |  |  |  |  |  |  |  |  | *pho-11* | intestinal acid PHOsphatase |
|  |  |  |  |  |  |  |  |  |  |  |  |  |  |  |  |  |  |  |  |  |  |  |  |  |  |  |  |  |  |  |  |  |  |  |  |  |  | *srt-30* | Serpentine Receptor, class T |
|  |  |  |  |  |  |  |  |  |  |  |  |  |  |  |  |  |  |  |  |  |  |  |  |  |  |  |  |  |  |  |  |  |  |  |  |  |  | Y119D3B.10 |  |
|  |  |  |  |  |  |  |  |  |  |  |  |  |  |  |  |  |  |  |  |  |  |  |  |  |  |  |  |  |  |  |  |  |  |  |  |  |  | *arf-1.1* | ADP-Ribosylation Factor related |

### Phenotypes enriched

|  |  |  |  |
| --- | --- | --- | --- |
| **Group name** | **Number in cluster** | **Enrichment** | **FDR corrected p** |
| amphid phasmid morphology variant | 23 | 32.64 | 1.24e-24 |
| amphid phasmid sensillum morphology variant | 23 | 32.64 | 1.24e-24 |
| sensillum morphology variant | 25 | 20.23 | 1.69e-21 |
| cilia morphology variant | 13 | 52.14 | 3.83e-16 |
| nervous system morphology variant | 26 | 10.90 | 1.58e-15 |
| osmotic avoidance defective | 14 | 37.99 | 3.66e-15 |
| osmotic avoidance variant | 14 | 35.88 | 9.17e-15 |
| ciliated neuron morphology variant | 13 | 42.83 | 9.43e-15 |
| chemotaxis variant | 19 | 15.93 | 9.33e-14 |
| aqueous positive chemotaxis variant | 16 | 22.71 | 9.68e-14 |
| dauer defective | 13 | 35.27 | 1.73e-13 |
| negative chemotaxis variant | 14 | 29.35 | 1.94e-13 |
| aqueous positive chemotaxis defective | 15 | 24.28 | 3.16e-13 |
| male mating efficiency reduced | 15 | 23.06 | 6.78e-13 |
| chemosensory response variant | 19 | 14.02 | 8.96e-13 |
| chemosensory behavior variant | 19 | 13.91 | 1.00e-12 |
| positive chemotaxis defective | 16 | 18.68 | 2.09e-12 |
| dauer formation variant | 16 | 17.78 | 4.49e-12 |
| aqueous chemosensory response variant | 16 | 17.78 | 4.49e-12 |
| dauer development variant | 16 | 17.57 | 5.43e-12 |
| positive chemotaxis variant | 16 | 16.40 | 1.65e-11 |
| ammonium chloride chemotaxis defective | 10 | 46.13 | 2.41e-11 |
| ammonium chloride chemotaxis variant | 10 | 46.13 | 2.41e-11 |
| cephalic sensillum morphology variant | 8 | 61.50 | 5.19e-10 |
| dauer arrest variant | 16 | 13.06 | 5.30e-10 |
| copulation variant | 17 | 11.28 | 9.71e-10 |
| axoneme morphology variant | 6 | 92.25 | 7.73e-09 |
| organ system morphology variant | 26 | 5.02 | 5.09e-08 |
| deirid sensillum morphology variant | 7 | 49.67 | 1.07e-07 |
| male ray morphology variant | 10 | 20.50 | 1.61e-07 |
| male nervous system morphology variant | 10 | 20.50 | 1.61e-07 |
| male response to contact defective | 7 | 43.05 | 3.60e-07 |
| odorant positive chemotaxis defective | 9 | 19.77 | 1.91e-06 |
| sodium chloride chemotaxis variant | 8 | 25.45 | 1.97e-06 |
| amphid morphology variant | 6 | 46.13 | 4.35e-06 |
| odorant positive chemotaxis variant | 9 | 17.67 | 5.24e-06 |
| sodium chloride chemotaxis defective | 7 | 30.75 | 5.24e-06 |
| axoneme short | 4 | 92.25 | 3.62e-05 |
| postembryonic development variant | 16 | 5.90 | 3.67e-05 |
| odorant chemosensory response variant | 9 | 13.61 | 4.72e-05 |
| chemical response variant | 19 | 4.65 | 7.06e-05 |
| vulva location variant | 6 | 27.68 | 1.25e-04 |
| male mating variant | 10 | 9.51 | 2.22e-04 |
| male behavior variant | 10 | 9.32 | 2.62e-04 |
| sexually dimorphic behavior variant | 10 | 9.04 | 3.34e-04 |
| post hermaphrodite contact variant | 8 | 12.72 | 4.13e-04 |
| male mating defective | 8 | 11.90 | 6.71e-04 |
| male morphology variant | 10 | 7.38 | 1.82e-03 |
| organism environmental stimulus response variant | 21 | 3.36 | 1.99e-03 |
| octopamine metabolism variant | 3 | 92.25 | 2.12e-03 |
| octopamine deficient | 3 | 92.25 | 2.12e-03 |
| amphid channel cilia morphology variant | 3 | 92.25 | 2.12e-03 |
| sexually dimorphic morphology variant | 10 | 7.21 | 2.19e-03 |
| morphology variant | 26 | 2.80 | 2.88e-03 |
| behavior variant | 24 | 2.76 | 8.31e-03 |
| organism behavior variant | 24 | 2.76 | 8.31e-03 |
| foraging behavior variant | 5 | 15.91 | 1.69e-02 |
| sodium chemotaxis defective | 4 | 23.06 | 2.52e-02 |
| cation positive chemotaxis variant | 4 | 19.42 | 4.65e-02 |
| cation positive chemotaxis defective | 4 | 19.42 | 4.65e-02 |

### Anatomy terms enriched

|  |  |  |  |
| --- | --- | --- | --- |
| **Group name** | **Number in cluster** | **Enrichment** | **FDR corrected p** |
| phasmid neuron | 42 | 18.28 | 1.11e-35 |
| phasmid sensillum | 42 | 16.92 | 1.74e-34 |
| amphid neuron | 47 | 9.30 | 3.64e-27 |
| amphid sensillum | 47 | 8.54 | 1.39e-25 |
| ciliated neuron | 48 | 7.63 | 3.82e-24 |
| inner labial neuron | 26 | 22.63 | 2.75e-23 |
| sensillum | 49 | 6.77 | 1.41e-22 |
| outer labial neuron | 22 | 30.29 | 2.21e-22 |
| inner labial sensillum | 26 | 18.45 | 5.21e-21 |
| labial sensillum | 26 | 16.89 | 5.38e-20 |
| outer labial sensillum | 22 | 23.06 | 1.53e-19 |
| PHAR | 17 | 19.60 | 3.58e-13 |
| PHAL | 17 | 19.60 | 3.58e-13 |
| PHA | 17 | 19.60 | 3.58e-13 |
| ASJR | 15 | 23.86 | 1.05e-12 |
| ASJL | 15 | 23.45 | 1.29e-12 |
| ASJ | 15 | 23.45 | 1.29e-12 |
| PHBL | 16 | 19.95 | 1.65e-12 |
| PHBR | 16 | 19.95 | 1.65e-12 |
| PHB | 16 | 19.95 | 1.65e-12 |
| sensory neuron | 31 | 6.00 | 1.38e-11 |
| phasmid right sensillum | 17 | 14.52 | 3.83e-11 |
| phasmid left sensillum | 17 | 14.52 | 3.83e-11 |
| ASKR | 15 | 17.30 | 1.18e-10 |
| ASKL | 15 | 17.08 | 1.37e-10 |
| ASK | 15 | 17.08 | 1.37e-10 |
| ASIR | 16 | 14.76 | 1.77e-10 |
| ASIL | 16 | 14.76 | 1.77e-10 |
| ASI | 16 | 14.76 | 1.77e-10 |
| postdeirid sensillum left | 11 | 30.75 | 3.98e-10 |
| postdeirid sensillum right | 11 | 30.75 | 3.98e-10 |
| postdeirid sensillum | 11 | 29.85 | 5.72e-10 |
| dopaminergic neuron | 16 | 12.72 | 1.61e-09 |
| chemosensory neuron | 22 | 7.52 | 2.10e-09 |
| ASHR | 14 | 15.75 | 2.46e-09 |
| ASHL | 14 | 15.75 | 2.46e-09 |
| ASH | 14 | 15.56 | 2.88e-09 |
| PDER | 10 | 31.81 | 3.19e-09 |
| PDEL | 10 | 31.81 | 3.19e-09 |
| PDE | 10 | 31.81 | 3.19e-09 |
| deirid sensillum | 12 | 20.50 | 4.49e-09 |
| odorsensory neuron | 16 | 11.18 | 9.90e-09 |
| glutamatergic neuron | 14 | 13.89 | 1.31e-08 |
| deirid neuron | 11 | 22.06 | 1.69e-08 |
| lumbar lateral left ganglion neuron | 19 | 8.00 | 2.79e-08 |
| ADLR | 13 | 14.99 | 2.88e-08 |
| ADLL | 13 | 14.99 | 2.88e-08 |
| ADL | 13 | 14.99 | 2.88e-08 |
| lumbar lateral right ganglion neuron | 19 | 7.79 | 4.18e-08 |
| lumbar neuron | 19 | 7.72 | 4.83e-08 |
| lumbar lateral left ganglion | 19 | 7.56 | 6.93e-08 |
| lumbar lateral ganglion | 19 | 7.40 | 9.75e-08 |
| lumbar lateral right ganglion | 19 | 7.40 | 9.75e-08 |
| R3AR | 11 | 18.45 | 1.17e-07 |
| R3AL | 11 | 18.45 | 1.17e-07 |
| R4AR | 11 | 18.12 | 1.39e-07 |
| R4AL | 11 | 18.12 | 1.39e-07 |
| R1AR | 11 | 18.12 | 1.39e-07 |
| R8AR | 11 | 17.80 | 1.56e-07 |
| R8AL | 11 | 17.80 | 1.56e-07 |
| R6AR | 11 | 17.80 | 1.56e-07 |
| R6AL | 11 | 17.80 | 1.56e-07 |
| R4BR | 11 | 17.80 | 1.56e-07 |
| R4BL | 11 | 17.80 | 1.56e-07 |
| R2AR | 11 | 17.80 | 1.56e-07 |
| R2AL | 11 | 17.80 | 1.56e-07 |
| R7AR | 11 | 17.50 | 1.68e-07 |
| R7AL | 11 | 17.50 | 1.68e-07 |
| R3BR | 11 | 17.50 | 1.68e-07 |
| R3BL | 11 | 17.50 | 1.68e-07 |
| R2BR | 11 | 17.50 | 1.68e-07 |
| R2BL | 11 | 17.50 | 1.68e-07 |
| R1BR | 11 | 17.50 | 1.68e-07 |
| R1BL | 11 | 17.50 | 1.68e-07 |
| OLLR | 10 | 21.45 | 1.68e-07 |
| OLLL | 10 | 21.45 | 1.68e-07 |
| OLL | 10 | 21.45 | 1.68e-07 |
| R7A | 11 | 17.50 | 1.68e-07 |
| head ganglion | 27 | 4.61 | 1.86e-07 |
| R8BR | 11 | 17.20 | 1.94e-07 |
| R8BL | 11 | 17.20 | 1.94e-07 |
| R6BR | 11 | 17.20 | 1.94e-07 |
| R6BL | 11 | 17.20 | 1.94e-07 |
| R9BR | 11 | 16.91 | 2.15e-07 |
| R9BL | 11 | 16.91 | 2.15e-07 |
| R9AR | 11 | 16.91 | 2.15e-07 |
| R9AL | 11 | 16.91 | 2.15e-07 |
| R7BR | 11 | 16.91 | 2.15e-07 |
| R7BL | 11 | 16.91 | 2.15e-07 |
| R5AR | 11 | 16.91 | 2.15e-07 |
| R5AL | 11 | 16.91 | 2.15e-07 |
| posterior lateral right ganglion | 11 | 16.91 | 2.15e-07 |
| lumbar ganglion | 19 | 6.90 | 2.15e-07 |
| R5A | 11 | 16.91 | 2.15e-07 |
| R9A | 11 | 16.91 | 2.15e-07 |
| OLQVR | 10 | 20.05 | 2.66e-07 |
| OLQVL | 10 | 20.05 | 2.66e-07 |
| OLQDR | 10 | 20.05 | 2.66e-07 |
| OLQDL | 10 | 20.05 | 2.66e-07 |
| OLQ | 10 | 20.05 | 2.66e-07 |
| R5BR | 11 | 16.37 | 2.89e-07 |
| R5BL | 11 | 16.37 | 2.89e-07 |
| ray 1 | 11 | 15.61 | 4.49e-07 |
| ray 1 right | 11 | 15.61 | 4.49e-07 |
| ray 1 left | 11 | 15.61 | 4.49e-07 |
| ray 3 | 11 | 15.61 | 4.49e-07 |
| ray 4 | 11 | 15.61 | 4.49e-07 |
| ray 3 left | 11 | 15.61 | 4.49e-07 |
| ray 3 right | 11 | 15.61 | 4.49e-07 |
| ray 4 left | 11 | 15.61 | 4.49e-07 |
| ray 4 right | 11 | 15.61 | 4.49e-07 |
| ray 2 | 11 | 15.38 | 5.06e-07 |
| ray 2 left | 11 | 15.38 | 5.06e-07 |
| ray 2 right | 11 | 15.38 | 5.06e-07 |
| ray 6 | 11 | 15.38 | 5.06e-07 |
| ray 8 | 11 | 15.38 | 5.06e-07 |
| ray 6 left | 11 | 15.38 | 5.06e-07 |
| ray 6 right | 11 | 15.38 | 5.06e-07 |
| ray 8 left | 11 | 15.38 | 5.06e-07 |
| ray 8 right | 11 | 15.38 | 5.06e-07 |
| ray 7 | 11 | 14.92 | 6.77e-07 |
| ray 9 | 11 | 14.92 | 6.77e-07 |
| ray 7 left | 11 | 14.92 | 6.77e-07 |
| ray 7 right | 11 | 14.92 | 6.77e-07 |
| ray 9 left | 11 | 14.92 | 6.77e-07 |
| ray 9 right | 11 | 14.92 | 6.77e-07 |
| outer labial lateral sensillum | 10 | 17.74 | 7.70e-07 |
| OL lateral right sensillum | 10 | 17.74 | 7.70e-07 |
| OL lateral left sensillum | 10 | 17.74 | 7.70e-07 |
| ray | 12 | 12.44 | 8.57e-07 |
| ray 5 | 11 | 14.50 | 8.97e-07 |
| ray 5 left | 11 | 14.50 | 8.97e-07 |
| ray 5 right | 11 | 14.50 | 8.97e-07 |
| ray neuron type A | 11 | 14.29 | 1.05e-06 |
| ray neuron type B | 11 | 14.09 | 1.22e-06 |
| amphid right sensillum | 19 | 5.86 | 2.45e-06 |
| amphid left sensillum | 19 | 5.82 | 2.72e-06 |
| ray neuron | 11 | 13.01 | 2.85e-06 |
| lateral ganglion | 20 | 5.26 | 5.36e-06 |
| OL quadrant ventral right sensillum | 10 | 13.98 | 7.85e-06 |
| OL quadrant dorsal right sensillum | 10 | 13.98 | 7.85e-06 |
| OL quadrant dorsal left sensillum | 10 | 13.98 | 7.85e-06 |
| outer labial quadrant sensillum | 10 | 13.77 | 9.01e-06 |
| OL quadrant ventral left sensillum | 10 | 13.77 | 9.01e-06 |
| lateral pharyngeal ganglion right neuron | 19 | 5.34 | 1.03e-05 |
| lateral pharyngeal ganglion right | 19 | 5.31 | 1.13e-05 |
| lateral pharyngeal ganglion left neuron | 19 | 5.31 | 1.13e-05 |
| neuron | 56 | 2.19 | 1.24e-05 |
| lateral pharyngeal ganglion left | 19 | 5.28 | 1.24e-05 |
| ganglion | 41 | 2.64 | 1.76e-05 |
| posterior lateral left ganglion | 11 | 10.57 | 2.38e-05 |
| posterior lateral ganglion | 11 | 10.46 | 2.65e-05 |
| ADFR | 9 | 14.07 | 4.38e-05 |
| tail neuron | 35 | 2.84 | 4.56e-05 |
| ADFL | 9 | 13.61 | 5.84e-05 |
| ADF | 9 | 13.61 | 5.84e-05 |
| ASER | 11 | 9.57 | 6.45e-05 |
| tail ganglion | 35 | 2.80 | 6.45e-05 |
| ASEL | 11 | 9.23 | 9.31e-05 |
| ASE | 11 | 8.82 | 1.46e-04 |
| male-specific | 13 | 6.85 | 1.76e-04 |
| R1AL | 8 | 13.92 | 2.77e-04 |
| ASGR | 8 | 12.95 | 4.76e-04 |
| ASGL | 8 | 12.95 | 4.76e-04 |
| ASG | 8 | 12.95 | 4.76e-04 |
| anterior pharyngeal ganglion (post) | 12 | 6.59 | 7.71e-04 |
| head neuron | 44 | 2.18 | 8.81e-04 |
| PQR | 6 | 17.30 | 2.56e-03 |
| AQR | 5 | 25.63 | 2.58e-03 |
| tail | 36 | 2.29 | 3.98e-03 |
| anterior ganglion | 12 | 5.27 | 6.75e-03 |
| embryonic cell | 20 | 3.27 | 7.05e-03 |
| head | 46 | 1.90 | 1.73e-02 |
| AWCR | 7 | 8.97 | 2.35e-02 |
| AWCL | 7 | 8.97 | 2.35e-02 |
| AWC | 7 | 8.97 | 2.35e-02 |
| nervous system | 44 | 1.91 | 2.39e-02 |
| ADER | 5 | 15.91 | 2.49e-02 |
| ADEL | 5 | 15.91 | 2.49e-02 |
| ADE | 5 | 15.91 | 2.49e-02 |
| body ganglion | 12 | 4.23 | 4.52e-02 |
| anterior deirid sensillum | 5 | 13.57 | 4.73e-02 |
| AWBR | 6 | 9.71 | 4.88e-02 |
| AWBL | 6 | 9.71 | 4.88e-02 |
| AWB | 6 | 9.71 | 4.88e-02 |

### GO terms enriched

|  |  |  |
| --- | --- | --- |
| **GO term** | **Number of genes** | **FDR-corrected p-value** |
| cilium | 28 | 2.0e-34 |
| cell projection organization | 26 | 4.1e-20 |
| cell part morphogenesis | 22 | 3.4e-19 |
| nonmotile primary cilium assembly | 12 | 6.7e-19 |
| intraciliary transport | 11 | 2.0e-18 |
| cell morphogenesis | 22 | 3.6e-18 |
| cytoskeleton-dependent intracellular transport | 11 | 1.2e-15 |
| microtubule-based movement | 14 | 1.1e-14 |
| ciliary transition zone | 9 | 4.7e-13 |
| nonmotile primary cilium | 11 | 3.5e-11 |
| anatomical structure formation involved in morphogenesis | 18 | 7.7e-10 |
| cell projection part | 10 | 2.2e-08 |
| cellular developmental process | 26 | 1.4e-07 |
| cellular component assembly | 19 | 4.2e-07 |
| guanyl nucleotide binding | 12 | 3.4e-06 |
| chemotaxis | 11 | 4.2e-06 |
| intracellular transport | 12 | 7.6e-06 |
| GTP binding | 11 | 2.4e-05 |
| response to external stimulus | 12 | 1.7e-04 |
| cellular component organization or biogenesis | 29 | 1.8e-04 |
| purine ribonucleotide binding | 20 | 1.4e-03 |
| GTPase activity | 7 | 1.6e-03 |
| carbohydrate derivative binding | 20 | 2.1e-03 |
| purine nucleoside binding | 19 | 4.0e-03 |
| ribonucleoside binding | 19 | 4.2e-03 |
| response to stimulus | 18 | 6.6e-03 |
| G-protein beta/gamma-subunit complex binding | 4 | 7.3e-03 |
| cellular process | 40 | 7.9e-03 |
| cellular localization | 13 | 1.0e-02 |
| enzyme linked receptor protein signaling pathway | 6 | 1.1e-02 |
| signaling | 23 | 1.1e-02 |
| G-protein coupled receptor binding | 4 | 1.4e-02 |
| dendrite | 5 | 1.5e-02 |
| regulation of transmembrane receptor protein serine/threonine kinase signaling pathway | 3 | 1.9e-02 |
| regulation of cellular response to growth factor stimulus | 3 | 1.9e-02 |
| adenylate cyclase-modulating G-protein coupled receptor signaling pathway | 4 | 1.9e-02 |
| nucleotide binding | 20 | 1.9e-02 |
| microtubule | 6 | 2.2e-02 |
| neuron projection | 5 | 2.4e-02 |
| heterotrimeric G-protein complex | 4 | 2.8e-02 |
| anion binding | 19 | 3.2e-02 |
| GTP catabolic process | 7 | 3.8e-02 |
| signal transduction | 19 | 4.2e-02 |
| guanosine-containing compound metabolic process | 7 | 4.4e-02 |
| extrinsic component of plasma membrane | 4 | 4.4e-02 |
| organelle organization | 18 | 4.8e-02 |

### Expression clusters enriched

|  |  |  |  |
| --- | --- | --- | --- |
| **Group name** | **Number in cluster** | **Enrichment** | **FDR corrected p** |
| Genes significantly enriched (> 2x, FDR < 5%) in a particular cell-type versus a reference sample of all cells at the same stage. WBPaper00037950:BAG-neuron\_embryo\_enriched | 81 | 16.46 | 2.66e-75 |
| Genes that show selective expression in a subset of cell types vs broadly expressed in many cell types. Correspond to 20% - 57% of enriched\_genes for a given cell type. WBPaper00037950:BAG-neuron\_embryo\_SelectivelyEnriched | 54 | 23.84 | 8.86e-57 |
| Candidate daf-19 down regulated genes with a statistically significant signal variation of 1.5-fold or greater. These were identified using a Significance Analysis of Microarrays (SAM). | 44 | 21.94 | 1.24e-43 |
| Candidate daf-19 down regulated genes with a statistically significant signal variation of 1.5-fold or greater. These were identified using a class comparisons tool from BRB Array Tools. | 57 | 9.72 | 2.58e-37 |
| Genes significantly enriched (> 2x, FDR < 5%) in a particular cell-type versus a reference sample of all cells at the same stage. WBPaper00037950:dopaminergic-neurons\_embryo\_enriched | 47 | 9.30 | 4.12e-29 |
| Genes predicted to be upregulated more than 2.0 fold in (AFD+AWB) datasets as compared to unsorted whole embryonic cells dataset. | 57 | 6.51 | 5.07e-28 |
| Genes significantly enriched (> 2x, FDR < 5%) in a particular cell-type versus a reference sample of all cells at both embryonic and larval stages. WBPaper00037950:dopaminergic-neurons\_CoreEnriched | 28 | 17.22 | 1.26e-23 |
| Genes that show selective expression in a subset of cell types vs broadly expressed in many cell types. Correspond to 20% - 57% of enriched\_genes for a given cell type. WBPaper00037950:dopaminergic-neurons\_embryo\_SelectivelyEnriched | 29 | 15.55 | 3.08e-23 |
| Embryonic Pan-neural Enriched Genes. | 70 | 3.99 | 1.06e-22 |
| Genes significantly enriched (> 2x, FDR < 5%) in a particular cell-type versus a reference sample of all cells at the same stage. WBPaper00037950:all-neurons\_larva\_enriched | 56 | 5.10 | 3.53e-22 |
| Genes that show selective expression in a subset of cell types vs broadly expressed in many cell types. Correspond to 20% - 57% of enriched\_genes for a given cell type. WBPaper00037950:all-neurons\_larva\_SelectivelyEnriched | 36 | 6.08 | 1.77e-15 |
| Larval Pan-neural Enriched Genes. | 58 | 3.46 | 5.27e-15 |
| Genes significantly enriched (> 2x, FDR < 5%) in a particular cell-type versus a reference sample of all cells at both embryonic and larval stages. WBPaper00037950:all-neurons\_CoreEnriched | 17 | 15.38 | 8.54e-13 |
| Genes significantly enriched (> 2x, FDR < 5%) in a particular cell-type versus a reference sample of all cells at the same stage. WBPaper00037950:all-neurons\_embryo\_enriched | 30 | 6.15 | 1.17e-12 |
| Genes that show selective expression in a subset of cell types vs broadly expressed in many cell types. Correspond to 20% - 57% of enriched\_genes for a given cell type. WBPaper00037950:all-neurons\_embryo\_SelectivelyEnriched | 17 | 14.52 | 2.23e-12 |
| WT-Pico Pan-neural Enriched Genes, with genes found multiple times in a single dataset removed (without dups). | 50 | 2.88 | 2.00e-09 |
| Genes that show selective expression in a subset of cell types vs broadly expressed in many cell types. Correspond to 20% - 57% of enriched\_genes for a given cell type. WBPaper00037950:dopaminergic-neurons\_larva\_SelectivelyEnriched | 25 | 5.01 | 2.10e-08 |
| Genes depleted in muscle cells (0hr muscle dataset). Dissociated myo-3::GFP embryos were cultured for 0 hours before FACS sorting. | 48 | 2.59 | 1.97e-07 |
| Genes significantly enriched (> 2x, FDR < 5%) in a particular cell-type versus a reference sample of all cells at the same stage. WBPaper00037950:dopaminergic-neurons\_larva\_enriched | 38 | 2.85 | 1.61e-06 |
| Total muscle depleted genes (complete list of non-overlapping genes from the 0hr and 24hr muscle depleted datasets). | 53 | 1.97 | 1.75e-04 |
| Genes that showed expression levels higher than the corresponding reference sample (embryonic 0hr reference). WBPaper00037950:BAG-neuron\_expressed | 79 | 1.59 | 5.66e-04 |
| Genes enriched in neuronal miRNA-induced silencing complexes (miRISC) as detected by immunoprecipitations and microarray analysis. | 19 | 2.53 | 4.11e-02 |

### Motifs enriched

|  |  |  |  |  |  |
| --- | --- | --- | --- | --- | --- |
| **Motif** | **Logo** | **Possible orthologs** | **Number of motifs in cluster** | **Enrichment** | **FDR corrected p** |
| RFX3\_1 |  | daf-19 (0.82) F52B5.7 | 61 | 14.09 | 1.5e-47 |
| pTH9223 |  | daf-19 (0.82) | 57 | 12.02 | 2.3e-40 |
| pTH9199 |  | daf-19 (0.82) | 58 | 9.89 | 1.2e-36 |
| V$ZID\_01 |  | ztf-28 | 128 | 1.26 | 2.2e-03 |
| MYF6\_f1 |  | hlh-1 | 44 | 1.87 | 3.1e-03 |
| TWST1\_f1 |  | hlh-15 K02D7.2 hlh-8 hlh-1 | 35 | 2.08 | 3.3e-03 |
| MYOD1\_f1 |  | hlh-14 hlh-1 | 22 | 2.58 | 5.7e-03 |
| Dmbx1\_2277 |  | alr-1 ceh-53 ceh-45 | 38 | 1.90 | 7.9e-03 |
| pTH10798 |  | Y75B8A.6 | 71 | 1.49 | 8.0e-03 |
| pTH6345 |  | lin-1 | 61 | 1.55 | 1.1e-02 |
| HepG2\_SRF\_HudsonAlpha |  | unc-120 | 144 | 1.17 | 1.1e-02 |
| NDF1\_f1 |  | ngn-1 hlh-32 | 48 | 1.67 | 1.5e-02 |
| CXXC1\_si |  | F52B11.1 | 15 | 2.93 | 1.8e-02 |
| pTH5539 |  | unc-120 | 141 | 1.16 | 2.4e-02 |
| V$DELTAEF1\_01 |  | ztf-6 | 121 | 1.22 | 2.4e-02 |
| pTH8556 |  | pax-2 (0.6) | 28 | 1.99 | 2.7e-02 |
| HLH27 |  | hlh-27 | 123 | 1.21 | 2.7e-02 |
| pTH9393 |  | ZC416.1 | 31 | 1.90 | 2.7e-02 |
| MA0067.1 |  | pax-1 | 20 | 2.31 | 3.2e-02 |
| Emx2\_3420 |  | ceh-2 | 52 | 1.55 | 3.4e-02 |
| Spdef |  | lin-1 | 134 | 1.18 | 3.4e-02 |
| MA0531.1 |  | Y5F2A.4 | 30 | 1.89 | 3.4e-02 |
| MA0229.1 |  | alr-1 lim-6 ceh-16 | 10 | 3.52 | 4.0e-02 |
| SP4\_f1 |  | klf-2 | 43 | 1.63 | 4.1e-02 |
| SRP000712\_Sox2 |  | sox-4 (0.8) | 107 | 1.24 | 4.8e-02 |

### Correlated (and anti-correlated) transcription factors

|  |  |
| --- | --- |
| **Transcription factor** | **Correlation** |
| ceh-57 | 0.93 |
| fkh-8 | 0.90 |
| dhhc-11 | 0.88 |
| nhr-51 | 0.88 |
| nhr-52 | 0.86 |
| nhr-258 | 0.85 |
| nhr-124 | 0.83 |
| C06E2.1 | 0.82 |
| daf-19 | 0.82 |
| sox-4 | 0.80 |
| Y22D7AL.16 | 0.79 |
| nhr-100 | 0.79 |
| vab-3 | 0.79 |
| nhr-277 | 0.77 |
| nhr-159 | 0.77 |
| nhr-158 | 0.76 |
| fozi-1 | 0.76 |
| nhr-138 | 0.73 |
| mbr-1 | 0.73 |
| ceh-58 | 0.73 |
| nhr-30 | 0.72 |
| C34F6.9 | 0.72 |
| nhr-167 | 0.71 |
| dro-1 | 0.71 |
| ceh-62 | 0.71 |
| nhr-173 | -0.38 |
| nhr-220 | -0.38 |
| ccch-5 | -0.39 |
| pes-1 | -0.39 |
| cep-1 | -0.39 |
| nhr-113 | -0.40 |
| sup-35 | -0.40 |
| Y57A10A.31 | -0.40 |
| lin-39 | -0.40 |
| nhr-76 | -0.40 |
| ccch-3 | -0.40 |
| attf-3 | -0.41 |
| snpc-1.2 | -0.41 |
| ceh-51 | -0.42 |
| nhr-246 | -0.42 |
| dmd-3 | -0.43 |
| sbp-1 | -0.43 |
| nhr-104 | -0.44 |
| nhr-122 | -0.44 |
| oma-2 | -0.45 |
| pal-1 | -0.45 |
| nhr-88 | -0.48 |
| nhr-261 | -0.53 |
| nhr-13 | -0.55 |
| hmbx-1 | -0.58 |

### ChIP peaks enriched

none found
